# Anti-Racism and Health Equity as Missing Values to Productive Aging: Implications for Emerging Professionals

**DOI:** 10.1093/geroni/igab046.1239

**Published:** 2021-12-17

**Authors:** Ernest Gonzales

**Affiliations:** New York University, New York, New York, United States

## Abstract

Productive aging scholarship has grown in scope and rigor over the last four decades, yet anti-racism and health equity have not been formally integrated into the conceptual framework. Furthermore, there is a dearth of research that explicates heterogeneity among a growing diverse older adult population. This presentation will integrate anti-racism and health equity as core values to productive aging scholarship in order to explore risk and protective factors to employment, volunteering, and caregiving among a growing diverse older adult population. Part of this presentation will include major findings from longitudinal population-based studies as well as key findings from a Consensus Statement by the National Academies of Sciences, Engineering, and Medicine (NASEM) on work and retirement trajectories. Dr. Gonzales will also share professional strategies (e.g., grant submissions, publishing, teaching) with ESPO members who want to center anti-racism, health equity, and social justice in their scholarship.

